# Stage-specific therapeutic strategies in eating disorders: translating clinical staging into tailored care pathways

**DOI:** 10.1192/j.eurpsy.2026.10377

**Published:** 2026-07-31

**Authors:** R. J. Bou Khalil

**Affiliations:** Psychiatry, Saint-Joseph university, Beirut, Lebanon

## Abstract

**Background:**

Eating disorders (EDs) are clinically heterogeneous and often evolve from early risk states to persistent, recurrent, and severe/enduring courses. While evidence-based treatments exist, outcomes remain variable, partly because most interventions are tested and delivered without systematic consideration of illness stage, medical risk, chronicity, and treatment history. Clinical staging offers a developmentally informed framework to align therapeutic intensity, targets, and outcomes with the patient’s position along the illness trajectory.

**Aims:**

This presentation proposes a pragmatic, stage-linked therapeutic framework for EDs to (1) match interventions to clinical need and risk, (2) reduce delays to effective care, and (3) support stepped, measurement-based treatment planning across settings.

**Approach:**

Drawing on transdiagnostic ED principles and staging approaches in psychiatry, I will map core interventions to illustrative stages (from risk/subthreshold presentations to severe and enduring illness), integrating medical stabilization, psychological mechanisms (e.g., cognitive rigidity, emotion dysregulation), family/system factors, and comorbidity.

**Key proposals:**

Early stages prioritize rapid access, engagement, brief evidence-based interventions, family involvement, and prevention of entrenchment. Established/recurrent stages emphasize multidisciplinary care, targeted psychotherapy (e.g., CBT-based approaches, family interventions where appropriate), relapse prevention, and optimized management of comorbid mood/anxiety, substance use, and personality pathology. Severe and enduring stages shift toward collaborative, recovery-oriented goals that balance medical safety with quality of life, functional restoration, and harm-reduction-informed decision-making when full symptom remission is not immediately attainable. Across stages, shared decision-making and measurement-based care are highlighted to guide step-up/step-down decisions and to operationalize stage transitions.

**Conclusions:**

A stage-informed therapeutic model can clarify “what works for whom, when,” improve resource allocation, and define stage-appropriate outcomes beyond symptom counts alone. Future directions include trials stratified by stage, validated staging indicators, and service models that embed early detection, stepped care, and continuity across transitions.

**Disclosure of Interest:**

None Declared

